# Identification and external validation of the hub genes associated with cardiorenal syndrome through time-series and network analyses

**DOI:** 10.18632/aging.203878

**Published:** 2022-02-08

**Authors:** Jingjing Liang, Xiaohui Huang, Weiwen Li, Yunzhao Hu

**Affiliations:** 1 Department of Cardiology, Shunde Hospital of Southern Medical University, Foshan 528000, China; 2 The Second School of Clinical Medicine, Southern Medical University, Guangzhou 510515, China

**Keywords:** cardiorenal syndrome, FN1, POSTN, diagnosis, biomarker

## Abstract

Cardiorenal syndrome (CRS), defined as acute or chronic damage to the heart or kidney triggering impairment of another organ, has a poor prognosis. However, the molecular mechanisms underlying CRS remain largely unknown. The RNA-sequencing data of the left ventricle tissue isolated from the sham-operated and CRS model rats at different time points were downloaded from the Gene Expression Omnibus (GEO) database. Genomic differences, protein–protein interaction networks, and short time-series analyses, revealed fibronectin 1 (FN1) and periostin (POSTN) as hub genes associated with CRS progression. The transcriptome sequencing data of humans obtained from the GEO revealed that FN1 and POSTN were both significantly associated with many different heart and kidney diseases. Peripheral blood samples from 20 control and 20 CRS patients were collected from the local hospital, and the gene expression levels of FN1 and POSTN were detected by real-time quantitative polymerase chain reaction. FN1 (area under the curve [AUC] = 0.807) and POSTN (AUC = 0.767) could distinguish CRS in the local cohort with high efficacy and were positively correlated with renal and heart damage markers, such as left ventricular ejection fraction. To improve the diagnostic ability, diagnosis models comprising FN1 and POSTN were constructed by logistic regression (F-Score = 0.718), classification tree (F-Score = 0.812), and random forest (F-Score = 1.000). Overall, the transcriptome data of CRS rat models were systematically analyzed, revealing that FN1 and POSTN were hub genes, which were validated in different public datasets and the local cohort.

## INTRODUCTION

Chronic heart and kidney disease often occur together and promote each other, leading to progressive deterioration of heart and renal function. The phenomenon in which acute or chronic impairment of the heart or kidney causes dysfunction of another organ was first defined as cardiorenal syndrome (CRS) by Ronco et al. in 2008 [1]. Several evidence-based medicine studies have shown that renal insufficiency is a vital prognostic predictor and risk factor for heart diseases, while cardiovascular deaths account for the largest proportion of renal diseases [2–4]. Microalbuminuria, a hallmark of renal dysfunction, is widely accepted as a predictive factor of cardiovascular events [5–7]. Additionally, patients with chronic heart failure are prone to microalbuminuria and exhibit unfavorable clinical outcomes [8, 9]. In general, a bidirectional relationship between heart and renal diseases has been confirmed.

Several mechanisms have been proposed to explain CRS pathophysiology. From a hemodynamic standpoint, the reduction of cardiac output caused by heart failure directly leads to a decrease in renal blood flow, causing renal ischemia. The decrease in renal blood flow could also activate the renin-angiotensin-aldosterone system (RAAS), thereby impairing the heart and renal functions [10]. From a physiological perspective, inflammation and oxidative stress play essential roles in CRS progression. The overactivation of RAAS promotes the release of inflammatory factors, such as interleukin 6, tumor necrosis factor α, and transforming growth factor (TGF), causing kidney fibrosis and ventricular remodeling [11, 12]. The upregulation of RAAS could further advance the production of reactive oxygen species (ROS), and excessive ROS leads to necrosis of renal and cardiac cells [13]. In addition to these classical hypotheses, other factors such as activation of the sympathetic nervous system, accumulation of uremic toxins, and endoplasmic reticulum stress are known to impact CRS [14, 15]. However, our current understanding of the initiation and development of CRS remains insufficient.

The rapid development of gene sequencing technology and big-data analysis has allowed further clarification of the latent molecular mechanisms underlying CRS. Chen et al. performed transcriptome sequencing of the right ventricle and kidney isolated from CRS mouse models and established lncRNA-miRNA-mRNA competing endogenous RNA networks, thus clarifying the comprehensive regulatory relationships of CRS [16]. Chuppa et al. utilized RNA-sequencing (RNA-seq) to analyze the left ventricle tissue of CRS rat models. They found that miR-21-5p could improve cardiac function by regulating peroxisome proliferator-activated receptor alpha [17]. These efforts broaden our horizons and highlight potential therapeutic targets for CRS. Nevertheless, the number of genome-wide studies on CRS remains limited.

Herein, the transcriptome data of the rat ventricle tissue at weeks 2, 4, 5, and 7 after subtotal nephrectomy were retrieved from the Gene Expression Omnibus (GEO) database. Genomic divergence, protein–protein interaction (PPI) network, and time-series analyses were conducted to identify the hub genes involved in CRS progression. Transcriptome sequencing of different types of heart and renal diseases downloaded from the GEO database was used for preliminary verification. The peripheral blood of the control and CRS subjects was also collected from the Shunde Hospital of Southern Medical University, and real-time quantitative polymerase chain reaction (RT-qPCR) was performed to detect gene expression levels. Finally, multiple machine learning algorithms were used to improve the diagnostic ability based on these hub genes.

## MATERIALS AND METHODS

### Data collection

The GSE98520 dataset, including the RNA-seq data with fragments per kilobase million format (FPKM) of the left ventricle tissue isolated from the sham-operated and treated rats at weeks 2, 4, 5, and 7 after the 5/6 nephrectomy, was directly obtained from the GEO database (https://www.ncbi.nlm.nih.gov/geo/) as the training dataset. The GEO datasets GSE2240, GSE161472, GSE36961, GSE66494, GSE125779, and GSE36961, containing different heart disease or kidney disease samples, were also downloaded. Detailed information on the public datasets utilized in this study is presented in Table 1. According to the platform annotation files collected from the GEO database, all probe IDs were transformed into gene symbols using R software (version 3.6.3). If multiple probes corresponded to a gene, an average value was adopted. Probes corresponding to multiple genes were excluded from analyses.

**Table 1:** The detailed information of the public datasets used in this study.

**ID**	**Platform**	**Organism**	**Tissue**	**Disease type**	**Control (*n*)**	**Disease (*n*)**	**References**
GSE98520	GPL14844	Sprague Dawley rat	Myocardium	Cardiorenal syndrome	8	8	[17]
GSE2240	GPL97	Human	Myocardium	Atrial fibrillation	20	10	[53, 54]
GSE161472	GPL11154	Human	Myocardium	Heart failure	37	47	[55]
GSE36961	GPL15389	Human	Myocardium	Hypertrophic cardiomyopathy	39	106	Unknown
GSE66494	GPL6480	Human	Kidney	Chronic kidney disease	8	53	[56]
GSE125779	GPL17586	Human	Kidney	Focal segmental glomerulosclerosis	8	8	[57]
GSE37171	GPL570	Human	Peripheral blood	Uremia	40	75	[58]

### Genomic difference analysis and PPI network construction

Genomic differences were analyzed to detect the differentially expressed genes between the sham-operated and subtotal nephrectomy rats at weeks 2, 4, 5, and 7 using the limma package. The filtering threshold to identify associated genes was set at *P* < 0.05. Genes exhibiting significant expression differences at all four time points were selected and included in the PPI network analysis [18]. The PPI network was based on the STRING database (version 11.5, https://cn.string-db.org/), and the confidence level was set to 0.4 [19]. The CytoHubba plug-in (version 0.1) in the Cytoscape software (version 3.8.0) was used to measure the importance of the genes in the network, and genes with a degree ≥10 were considered as hub genes [20].

### Time-series analysis

Time-series analysis was performed using the Short Time-series Expression Miner (STEM, version 1.3.13). The data were normalized to the expression values of the sham-operated samples. The STEM clustering method was utilized to conduct the clustering with the following parameters: the maximum number of model profiles = 50 and maximum unit change in model profiles between time points = 2 [21]. The advance options were all set to the default values.

### Functional enrichment analysis

Gene Ontology (GO) functional annotation was conducted using the clusterProfiler package after transforming the gene symbols into Entrez IDs according to the org.Rn.eg.db or org.Hs.eg.db package. Terms with *P* < 0.05 and Q < 0.05 were considered statistically significant.

### Clinical samples

The study protocol was reviewed and approved by the Ethics Committee of the Shunde Hospital of Southern Medical University (Ethics Approval Number: 20210207). All participants signed an informed consent form. Peripheral blood samples (2 mL) of 20 control and 20 CRS subjects were collected within 24 h of admission and stored in EDTA anticoagulant tubes at 4°C. CRS diagnosis was based on the latest clinical guidelines [22, 23]. The control subjects were defined as those without CRS, severe cardio or renal dysfunction, malignant tumors, severe infection, or other factors which could possibly influence the gene expression level. The clinicopathological features of patients, including age, sex, smoking, diabetes history, left ventricular ejection fraction (LVEF), N-terminal pro-B-type natriuretic peptide (NTproBNP), serum creatinine (Scr), blood urea nitrogen (BUN), and uric acid (UA), were also recorded.

### RT-qPCR

Total RNA was extracted using the Trizol-chloroform method (Trizol reagent, Sigma-Aldrich, China) after the red blood cells of the whole blood sample were lysed with erythrocyte lysis buffer (Sigma-Aldrich, Saint Louis, MO, USA) for 15 min at room temperature. The purity and concentration of the RNA were measured using a Nanodrop2000 spectrophotometer (Thermo Scientific, Waltham, MA, USA). Complementary DNA was synthesized and amplified using the PrimeScript RT Reagent Kit (Takara, Dalian, China) and SYBR Premix ExTaq kit (Takara, China). The PCR experiments were conducted on an Applied Biosystems 7600 thermocycler (ABI, USA). Gene expression levels were normalized to GAPDH, and the 2^−ΔΔCt^ method was used to calculate the definite RNA expression values. The primer sequences used in this study are listed in Table 2.

**Table 2:** The primer sequence used in this study.

**Gene**	**Primer sequence (5′–3′)**
FN1	Forward: CGGTGGCTGTCAGTCAAAG
	Reverse: AAACCTCGGCTTCCTCCATAA
POSTN	Forward: CTCATAGTCGTATCAGGGGTCG
	Reverse: ACACAGTCGTTTTCTGTCCAC
GAPDH	Forward: GGAGCGAGATCCCTCCAAAAT
	Reverse: GGCTGTTGTCATACTTCTCATGG

### Construction of diagnostic models based on machine learning algorithm

To improve the diagnostic ability of CRS, we constructed diagnostic models based on the screened core genes. Logistic regression was performed to establish the diagnostic model, and a nomogram was drawn to visualize the model using the rms package [24]. The classification tree was constructed using the rpart package [25], and the random forest model was developed using the randomForest package with the following parameters: 500 as the ntree and 3 as the mtry [26]. The confusion matrices, accuracy, precision, recall, and F-Score were used to measure the predictive ability of each machine learning diagnosis model.

### Identification of the functionally-related genes

The top 20 functionally related genes of the hub genes were identified using the GeneMANIA database (http://genemania.org/search/homo-sapiens/). The number of the maximum resultant genes was set to 20, and that of maximum resultant attributes was set to 10. The query-dependent weighting method was chosen automatically by the website [27].

### Statistical analysis

Statistical analysis were performed using R (version 3.6.3) and GraphPad Prism (version 8.4.3). Data are presented as mean ± standard deviation (SD). Student’s *t*-test was used to compare gene expression differences in the PCR experiments. The Welch-corrected *t*-test was adopted to compare the differences in gene expression levels between the control and disease groups obtained from the GEO database and clinicopathological parameters of the control and CRS subjects from the Shunde Hospital of Southern Medical University. The association between the hub gene expression level and clinicopathological variables was measured using the Spearman correlation test. The receiver operating curve (ROC) and corresponding area under curve (AUC) were obtained from the pROC package. Unless otherwise specified, *P* < 0.05 was considered to be statistically significant; ^*^P < 0.05, ^**^P < 0.01, ^***^P < 0.001.

## RESULTS

### Genomic difference analysis and PPI network construction

**Figure 1:**
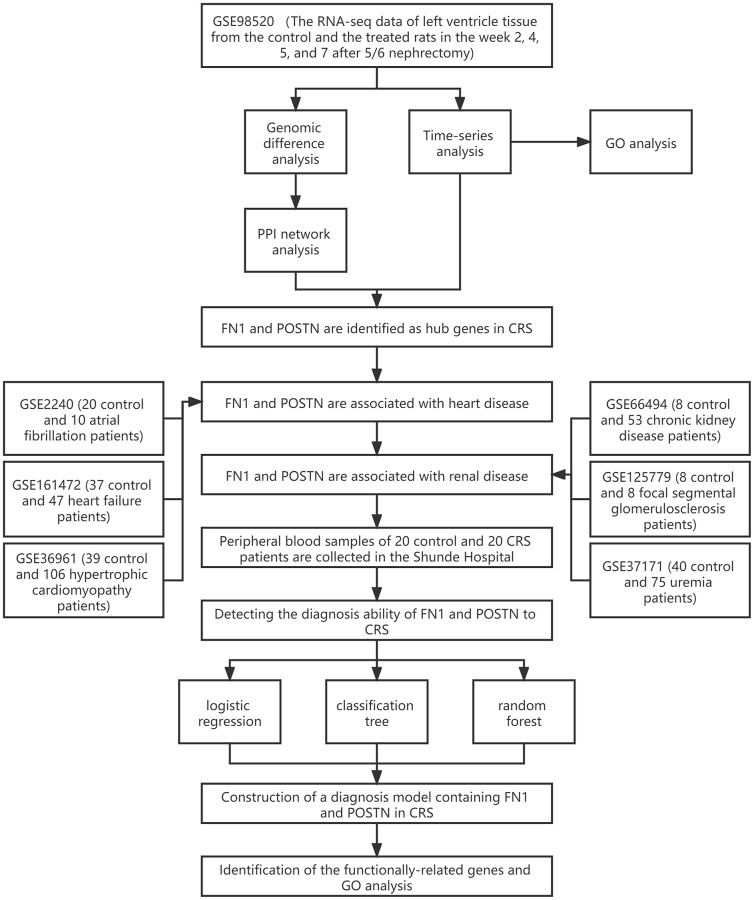
The workflow of the present study.

**Figure 2:**
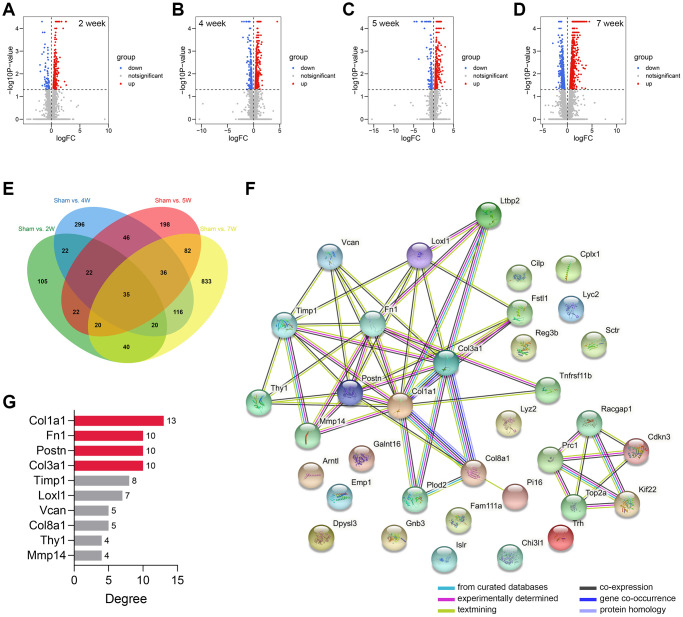
**Genomic difference analyses and PPI network construction.** (**A**–**D**) The volcano plots showing the differentially-expressed genes between the sham-operated and CRS model rats in week 2 (**A**), week 4 (**B**), week 5 (**C**), and week 7 (**D**). (**E**) A sum of 35 differentially expressed genes were overlapped. (**F**) The PPI network of the 35 genes. (**G**) The Top 10 genes with the highest degree value in the network. Abbreviation: PPI: protein-protein interaction network.

The workflow of the study protocol is shown in Figure 1. The R code used in this study is presented in Supplementary Material. The differentially expressed genes between the sham-operated and CRS model rats were analyzed. A total of 286 (Figure 2A), 593 (Figure 2B), 463 (Figure 2C), and 1182 (Figure 2D) genes showing expression differences were screened in 2-, 4-, 5-, and 7-week old rats, respectively, and 35 genes were found to overlap (Figure 2E). Subsequently, 35 genes were included in the PPI network (Figure 2F). The CytoHubba app revealed that the degrees of Col1a1, FN1, POSTN, and Col3a1 were greater than or equal to 10, and thus, the four genes were selected for subsequent analysis (Figure 2G, Supplementary Table 1).

### Time-series analysis and functional annotation

**Figure 3:**
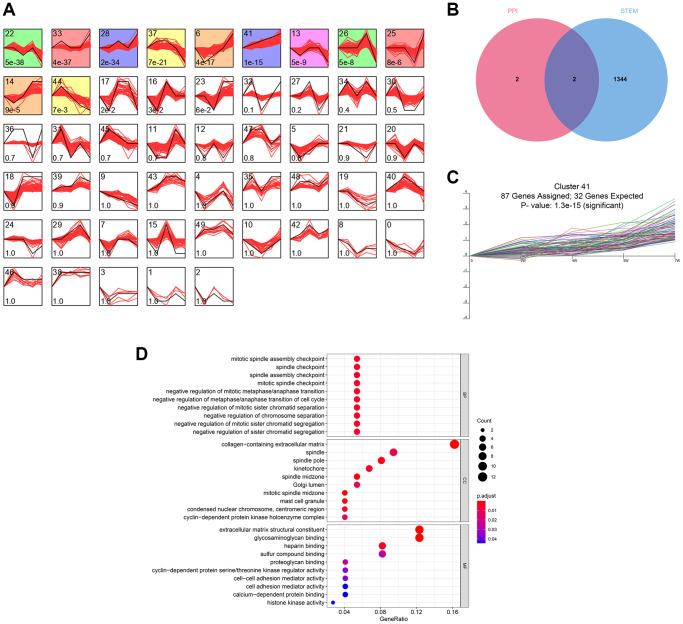
**The time-series analyses.** (**A**) 11 gene clusters were identified. The number at bottom-left in each cell represent the *P*-value and the number at top-left is the gene cluster ID. (**B**) FN1 and POSTN were con-determined by the network analysis and the time-series analysis. (**C**) FN1 and POSTN were encompassed in cluster 41, of which the *P*-value <0.001. (**D**) The GO functional annotation of the gene cluster 41. Abbreviation: GO: gene ontology.

The time-series analysis of the transcriptome sequencing data of the CRS model rats at 4 time nodes identified 11 different gene clusters (*P* < 0.05) (Figure 3A). A total of 1346 genes were included in these clusters, of which fibronectin 1 (FN1) and periostin (POSTN) were determined by PPI network analysis (Figure 3B). Interestingly, FN1 and POSTN were both members of cluster 41 (*P* < 0.001, Figure 3C). GO enrichment analysis indicated that the genes in cluster 41 were mainly involved in cell cycle- and immune-related pathways, such as negative regulation of metaphase/anaphase transition of the cell cycle, spindle checkpoint, and mast cell granules (Figure 3D). The synthesis of pro-inflammatory mediators always increases in CRS, causing cell death and fibrosis [28]. In addition, cell cycle arrest plays essential roles in pathological processes as the renal and cardiac cells usually undergo cell cycle arrest to prevent possible DNA damage from cell division when the cells undergo cellular stress [29]. Generally, these findings correspond to those of previous studies.

### FN1 and POSTN are associated with many different heart and renal diseases

**Figure 4:**
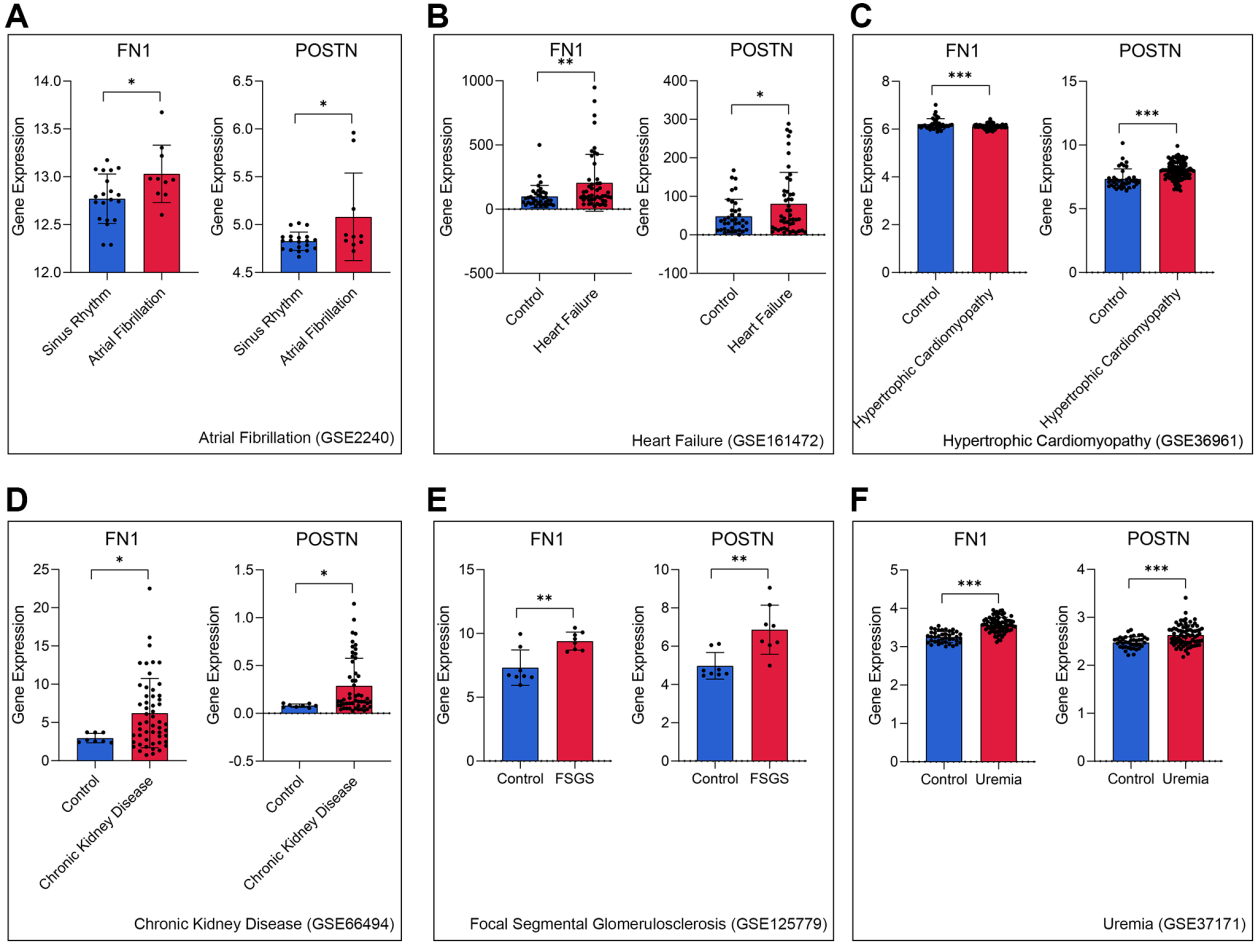
FN1 and POSTN were up-regulated in multiple heart disease and renal diseases samples, including atrial fibrillation (**A**), heart failure (**B**), hypertrophic cardiomyopathy (**C**), chronic kidney disease (**D**), focal segmental glomerulosclerosis (**E**), and uremia (**F**), compared with the control samples. Abbreviations: FSGS: focal segmental glomerulosclerosis. ^*^P < 0.05; ^**^P < 0.01; ^***^P < 0.001.

To further verify the role of FN1 and POSTN in CRS, transcriptome data of different heart and kidney diseases were downloaded. We found that FN1 was significantly upregulated in the atrial fibrillation (*P* < 0.05, Figure 4A), heart failure (*P* < 0.01, Figure 4B), hypertrophic cardiomyopathy (*P* < 0.001, Figure 4C), chronic kidney disease (*P* < 0.05, Figure 4D), focal segmental glomerulosclerosis (*P* < 0.01, Figure 4E), and uremia (*P* < 0.001, Figure 4F) samples compared with control samples. Similar trends were also observed in POSTN, as shown in Figure 4A–4F (*P* < 0.05). The indirect evidence partly demonstrated that FN1 and POSTN are strongly associated with many different heart and kidney diseases, thereby influencing the pathogenesis of CRS.

### FN1 and POSTN are promising diagnostic biomarkers of CRS

**Figure 5:**
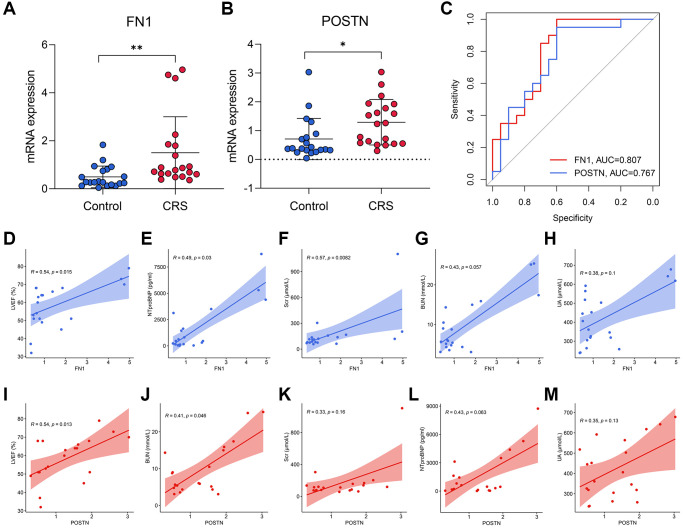
**The diagnostic ability of FN1 and POSTN to CRS.** (**A**, **B**) FN1 (**A**) and POSTN (**B**) were up-regulated in the peripheral blood sample of CRS patients from the Shunde Hospital of Southern Medical University. (**C**) The ROC analysis showed that FN1 and POSTN could discriminate the CRS samples with high efficacy. (**D**–**H**) The association of FN1 expression with LVEF (**D**), NTproBNP (**E**), Scr (**F**), BUN (**G**), and UA (**H**). (**I**–**M**) The association of POSTN expression with LVEF (**I**), BUN (**J**), Scr (**K**), NTproBNP (**L**), and UA (**M**). Abbreviations: CRS: cardiorenal syndrome; ROC: receiver operating curve; LVEF: left ventricular ejection fraction; NTproBNP: N-terminal-pro-B-type natriuretic peptide; Scr: serum creatinine; BUN: blood urea nitrogen; UA: uric acid. ^*^P < 0.05; ^**^P < 0.01; ^***^P < 0.001.

RT-qPCR was used to measure the expression levels of FN1 and POSTN in the peripheral blood of control and CRS patients. The original CT values of FN1, POSTN, and GAPDH are listed in Supplementary Table 2. The baseline clinicopathological information of the subjects enrolled in this study is displayed in Table 3. Except for smoking history (*P* < 0.05), Scr (*P* < 0.05), BUN (*P* < 0.01), and UA (*P* < 0.01), other parameters, including age, sex, diabetes history, LVEF, and NT-proBNP, revealed no significant differences between the control and CRS groups. Compared with the control subjects, the CRS cases exhibited higher expression levels of FN1 (*P* < 0.01, Figure 5A) and POSTN (*P* < 0.05, Figure 5B). ROC analysis indicated that FN1 (AUC = 0.807, Figure 5C) and POSTN (AUC = 0.767, Figure 5C) were both diagnostic biomarkers with high efficacy. The association of the expression values of FN1 and POSTN with routine laboratory tests was also detected. FN1 was significantly associated with LVEF (R = 0.54, *P* < 0.05, Figure 5D), NT-proBNP (R = 0.49, *P* < 0.05, Figure 5E), and Scr (R = 0.57, *P* < 0.01, Figure 5F), but no significant association was observed with BUN (R = 0.43, *P* = 0.057, Figure 5G) or UA (R = 0.38, *P* = 0.1, Figure 5H). POSTN was further significantly correlated with LVEF (R = 0.54, *P* < 0.05, Figure 5I) and BUN (R = 0.41, *P* < 0.05, Figure 5J), while no significant association was observed between POSTN and Scr (R = 0.33, *P* = 0.16, Figure 5K), NT-proBNP (R = 0.43, *P* = 0.063, Figure 5L), and UA (R = 0.35, *P* = 0.13, Figure 5M). The association of CRS with LVEF [30], NTproBNP [31], Scr [32], and BUN [33] has been verified in multiple studies, which strengthens the reliability of FN1 and POSTN.

**Table 3:** The baseline information of the 20 control and 20 CRS patients.

**Parameters**	**Control (*n* = 20)**	**CRS (*n* = 20)**	***P*-value**	**Significance**
Male (*n*, %)	13 (65.0%)	15 (75.0%)	0.302	ns
Age (years)	59.1 ± 16.8	55.1 ± 16.8	0.450	ns
Smoking (*n*, %)	6 (30.0%)	11 (55.0%)	0.015	^*^
Diabetes history (*n*, %)	4 (20.0%)	8 (40.0%)	0.068	ns
LVEF (%)	64.2 ± 7.7	58.3 ± 12.2	0.078	ns
NTproBNP (pg/ml)	1474.6 ± 2457.9	1580.7 ± 2317.9	0.889	ns
Scr (μmol/L)	52.1 ± 20.3	168.4 ± 227.7	0.029	^*^
BUN (mmol/L)	4.8 ± 1.2	9.6 ± 6.9	0.004	^**^
UA (μmol/L)	309.4 ± 81.6	419.7 ± 143.6	0.005	^**^

Abbreviations: CRS: cardiorenal syndrome; LVEF: left ventricular ejection fraction; NTproBNP: N-terminal-pro-B-type natriuretic peptide; Scr: serum creatinine; BUN: blood urea nitrogen; UA: uric acid; ns: not significance. ^*^P < 0.05; ^**^P < 0.01; ^***^P < 0.001.

### Construction of the diagnostic models based on FN1 and POSTN

**Figure 6:**
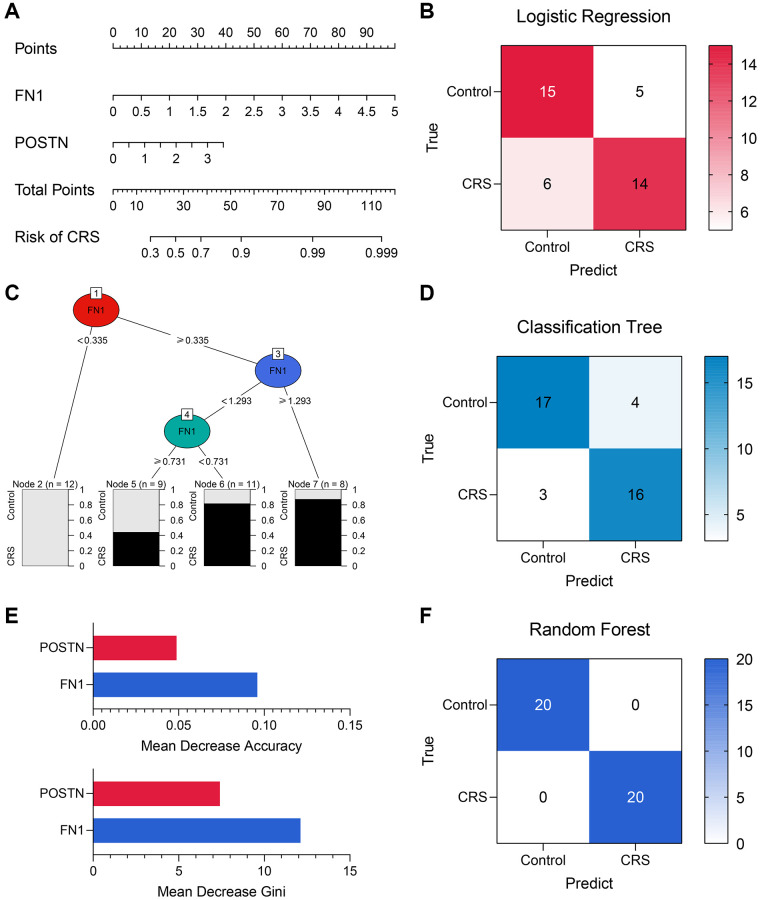
**The machine learning models encompassing FN1 and POSTN to diagnose CRS.** (**A**) A nomogram was drawn to visualize the logistic regression model. (**B**) The confusion matrix showed the predictive performance of the logistic regression model. (**C**) The classification tree was established to diagnose CRS. (**D**) The confusion matrix of the classification tree model. (**E**) The mean decrease accuracy and mean decrease Gini of the features in the random forest model. (**F**) The confusion matrix exhibited that the random forest model could distinguish the CRS samples with high efficacy. Abbreviation: CRS: cardiorenal syndrome.

Diagnostic models containing FN1 and POSTN were established using multiple machine learning algorithms. The logistic regression model was constructed as follows: logistic score = −2.10 + 1.58^*^EXP(FN1) + 0.88^*^EXP(POSTN), where EXP represented the mRNA expression level of FN1 or POSTN. A nomogram including FN1 and POSTN was constructed to help clinicians better understand the logistic model (Figure 6A). The confusing matrix of indicated that the logistic regression model could predict CRS with high efficacy (Figure 6B). The classification tree is shown in Figure 6C. Compared with the logistic regression model, the classification tree showed a higher predictive ability (Figure 6D). Unexpectedly, it was found that only FN1 was included in the classification tree model, implying that FN1 had better predictive ability than POSTN. In the random forest model, the importance of FN1 and POSTN was quantified as a mean decrease in accuracy and Gini, which were previously described [34]. Compared with POSTN, FN1 exhibited higher mean decreases in accuracy and Gini (Figure 6E), which was in agreement with the classification tree analysis. The random forest model could distinguish the CRS sample with the highest effectiveness (Figure 6F, Supplementary Figure 1A and 1B), which is in alignment with the results of several previous studies that have provided proof of its strong performance [35–37]. The accuracy, precision, recall, and F-score of each model are listed in Table 4. In addition, the predictive ability of CRS for clinicopathological features and established diagnostic models were also compared (Supplementary Figure 1A and 1B). Overall, compared with the traditional biomarkers or single biomarkers like FN1 and POSTN, the combination of FN1 and POSTN through machine learning algorithms, especially random forest, greatly improved the efficiency of CRS diagnosis.

**Table 4:** The performance of the logistic regression model, the classification tree model, and the random forest model.

**Model**	**Accuracy**	**Precision**	**Recall**	**F-Score**
Logistic regression model	0.725	0.737	0.700	0.718
Classification tree model	0.825	0.800	0.842	0.812
Random forest model	1.000	1.000	1.000	1.000

### Functionally-related genes of FN1 and POSTN

**Figure 7:**
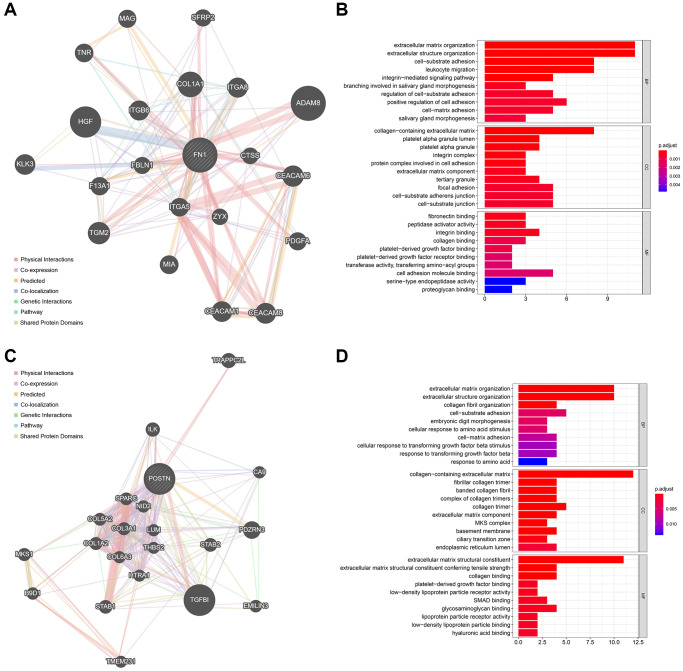
**The functionally-related genes of FN1 and POSTN.** (**A**) The Top 20 genes showing the highest association with FN1. (**B**) GO functional analysis of the FN1-related genes. (**C**) The Top 20 genes most associated with POSTN. (**D**) GO enrichment analysis of the POSTN-related genes. Abbreviation: GO: gene ontology.

The top 20 most strongly associated genes of FN1 and their interaction relationships are illustrated in Figure 7A. The size of the gene nodes represents the importance of the genes in the network, and the thickness of the lines is positively correlated with the interaction strength. GO analyses revealed that FN1 and its associated genes mostly participate in extracellular matrix-, immune-, and platelet-related biological processes (Figure 7B). The top 20 POSTN-related genes are shown in Figure 7C, and their functions were mainly associated with the extracellular matrix, amino acids, and TGF (Figure 7D). These findings indicate the possible mechanisms by which FN1 and POSTN are involved in the bidirectional interaction between the heart and kidney.

## DISCUSSION

CRS is a complex heart–kidney disease characterized by high morbidity and mortality, resulting in a tremendous social burden worldwide [38]. About 20–40% of patients with acute heart failure suffer from kidney dysfunction, and almost 40–60% of patients with chronic heart failure experience chronic kidney disease [39, 40]. Exploration of the molecular mechanisms in CRS is critical and difficult. The advancement and popularization of gene sequencing technology has provided an opportunity to further elucidate the pathological processes of this disease. Recently, many novel biomarkers associated with CRS have been reported, including cystatin 3, galectin 3, NGAL, and KIM1 [31, 41]. These biomarkers not only provide the clinical guidelines for CRS diagnosis and prognosis, but also suggest the underlying cut-in points for mechanistic studies. However, given the complexity of CRS, the current findings are far from sufficient.

The present study systematically analyzed the transcriptomic data of the rat ventricle tissue at weeks 2, 4, 5, and 7 after subtotal nephrectomy through genomic difference detection, PPI network analysis, and time-series analysis; FN1 and POSTN were ultimately identified as hub genes associated with CRS. The validation in different public datasets and local clinical samples indicated that FN1 and POSTN were both significant diagnostic biomarkers for CRS, which verified the findings from the animal experiments. Here, we report that levels of FN1 and POSTN were obviously increased not only in the diseased heart and kidney, but also in the plasma of CRS patients, implying that FN1 and POSTN are underlying cardiorenal connectors. FN1, encoding fibronectin, a protein located in the plasma, cell surface, and extracellular matrix, is mainly involved in cell adhesion and migration [42]. POSTN is also located in the extracellular space and regulates tissue development and regeneration. The vital roles of FN1 and POSTN in heart and kidney disease have previously been reported. Wang et al. found that FN1 was associated with immune infiltration in diabetic nephropathy [43], Su et al. reported that FN1 could regulate the process of renal fibrosis [44], Zhao et al. disclosed that FN1 was involved in rat H9C2 cardiomyocyte growth by regulating cell cycle arrest [45], and Patel et al. discovered that the expression of FN1 increased in patients with sudden cardio death [46]. Cardiofibrosis has a significant influence on the prognosis of heart diseases, which can be regulated by POSTN [47, 48]. POSTN was also found to be associated with renal diseases, including diabetic kidney disease [49], immunoglobulin A nephropathy [50], and polycystic kidney disease [51]. Hence, regardless of the absence of a regulatory relationship of POSTN and FN1 with CRS, FN1 and POSTN may exert important biological functions in the pathogenesis of CRS. Time-series analysis indicated that FN1 and POSTN were both members of cluster 41, and were thus mainly associated with immune-related pathways, which corresponded to the enrichment results of their functionally related genes. Immune-mediated dysregulation is well known for its vital role in CRS development, directly evidenced by the strong upregulation of plasma proinflammatory cytokines in CRS patients [52]. In summary, FN1 and POSTN are latent cardiorenal connectors that may function by regulating the immune response in CRS.

Another highlight of this study is the establishment of CRS diagnostic models comprising FN1 and POSTN. To ameliorate the predictive ability, the diagnostic models were constructed using three different machine learning algorithms, namely logistic regression, classification tree, and random forest. The ROCs revealed that the random forest model could distinguish CRS samples from control samples with high efficacy. Compared with traditional biomarkers, the performance of the random forest model is more satisfactory. In general, novel genetic diagnostic tools based on FN1 and POSTN are presented, providing new choices for clinicians.

The limitations of this study should not be neglected. First, we only performed the direct validation of the diagnostic value of FN1 and POSTN in the local hospital, and external verification in other centers would be beneficial to further clarify the clinical usefulness of these biomarkers. Second, we revealed that FN1 and POSTN serve as novel biomarkers of CRS in animal experiments and different cohorts, but how they affect the progression of CRS remains unclear. Further experiments are needed to explore the processes by which FN1 and POSTN affect the development of CRS.

In summary, the transcriptome sequencing data of the CRS rat models at different time models were systematically analyzed, and FN1 and POSTN were thus identified as novel biomarkers in CRS. These were externally validated in public datasets and local clinical samples, providing novel insights into the molecular mechanisms of CRS.

## Supplementary Materials

Supplementary Material

Supplementary Figure 1

Supplementary Tables
