# Supplementary material for: Identification and external validation of the hub genes associated with cardiorenal syndrome through time-series and network analyses
Source: Aging (Albany NY). 2022 Feb 8;14(3):1351–73. doi: 10.18632/aging.203878 (PMC8876909; doi:10.18632/aging.203878)
Supplement: Supplementary Material [file aging-14-203878-s001.pdf]

SUPPLEMENTARY MATERIAL

The R code used in this study

```
#Genomic difference analysis
library(limma)
logFoldChange=0
adjustP=0.05
rt=read.table("GSE98520_Supplemental_Table_1_5-6Nx_vs._Sham_Wk2.txt",sep="\
t",header=T,check.names=F)
rt=as.matrix(rt)
rownames(rt)=rt[,1]
exp=rt[,2:ncol(rt)]
dimnames=list(rownames(exp),colnames(exp))
rt=matrix(as.numeric(as.matrix(exp)),nrow=nrow(exp),dimnames=dimnames)
rt=avereps(rt)
modType=c(rep("con",conNum),rep("treat",treatNum))
design <- model.matrix(~0+factor(modType))
colnames(design) <- c("con","treat")
fit <- lmFit(rt,design)
cont.matrix<-makeContrasts(treat-con,levels=design)
fit2 <- contrasts.fit(fit, cont.matrix)
fit2 <- eBayes(fit2)
allDiff=topTable(fit2,adjust='fdr',number=200000)
write.table(allDiff,file="All_limma.xls",sep="\t",quote=F)
#The volcano plot
library(ggpubr)
library(ggthemes)
deg.data <- read.table("score.cor.txt", header = T, sep = "\t")
head(deg.data)
deg.data$logp <- -log10(deg.data$FDR)
deg.data$group = "notsignificant"
deg.data$group[which((deg.data$FDR < 0.05) & (deg.data$logFC > 0))] = "up"
deg.data$group[which((deg.data$FDR < 0.05) & (deg.data$logFC < 0))] = "down"
table(deg.data$group)
deg.data$label = ""
deg.data <- deg.data[order(deg.data$FDR),]
up.genes <- head(deg.data$gene[which(deg.data$group == "up")], 0)
down.genes <- head(deg.data$gene[which(deg.data$group == "down")], 0)
deg.top10.genes <- c(as.character(up.genes),as.character(down.genes))
deg.data$label[match(deg.top10.genes, deg.data$gene)] <- deg.top10.genes
pdf(file="volcano.pdf",
                    width = 5,
                    height = 4,
)
ggscatter(deg.data, x = "logFC", y = "logp", color = "group", palette = c("#4169E1","#BBBBBB","#E3170D"), size
= 1, label = deg.data$label, font.label = 8, repel = T, xlab = "Spearman Correlation", ylab = "-log10P",) +
theme_base() +

geom_hline(yintercept = 1.30, linetype = "dashed") + geom_vline(xintercept = c(0,0), linetype = "dashed")
dev.off()
#the differentially expressed genes between the sham-operated and subtotal nephrectomy rats in week 4, 5, and 7 are
obtained using the same algorithms.

#The protein-protein interaction network was constructed in STRING database.
#The time-series analyses were conducted by the Short Time-series Expression Miner  software.
```

```
#GO functional enrichment
library("org.Rn.eg.db")
rt=read.table("DEGs.txt",sep="\t",check.names=F,header=T)
genes=as.vector(rt[,1])
entrezIDs <- mget(genes, org.Rn.egSYMBOL2EG, ifnotfound=NA)
entrezIDs <- as.character(entrezIDs)
out=cbind(rt,entrezID=entrezIDs)
write.table(out,file="id.txt",sep="\t",quote=F,row.names=F)
library("clusterProfiler")
library("enrichplot")
library("ggplot2")
rt=read.table("id.txt",sep="\t",header=T,check.names=F)

rt=rt[is.na(rt[,"entrezID"])==F,]

gene=rt$entrezID
kk <- enrichGO(gene = gene,
               OrgDb = org.Rn.eg.db,
               pvalueCutoff =0.05,
               qvalueCutoff = 0.05,
               ont="all",
               readable =T)
write.table(kk,file="GO.txt", sep="\t",quote=F, row.names = F)

pdf(file="bubble_go.pdf",width = 10,height = 8)
dotplot(kk,showCategory = 10,split="ONTOLOGY") + facet_grid(ONTOLOGY~.,  scale='free')
dev.off()

#The calculation of the areas under the receiver operating curve.
library(pROC)
inputFile="input.txt"
outFile="ROC.pdf"

rt=read.table(inputFile,header=T,sep="\t",check.names=F,row.names=1)
y=colnames(rt)[1]
bioCol=c("#E3170D","#4169E1","#03A89E","#03A89E")
if(ncol(rt)>4){
     bioCol=rainbow(ncol(rt))}

pdf(file=outFile,width=4.5,height=4.5)
roc1=roc(rt[,y], as.vector(rt[,2]))
aucText=c( paste0(colnames(rt)[2],", AUC=",sprintf("%0.3f",auc(roc1))) )
plot(roc1, col=bioCol[1])
for(i in 3:ncol(rt)){
      roc1=roc(rt[,y], as.vector(rt[,i]))
      lines(roc1, col=bioCol[i-1])
      aucText=c(aucText, paste0(colnames(rt)[i],", AUC=",sprintf("%0.3f",auc(roc1))) )
}
legend("bottomright", aucText, lwd=2,bty="n", col=bioCol[1:(ncol(rt)-1)])  dev.off()

#The Spearman correlation analyses
library(ggplot2)
library(ggpubr)
rt <- read.table("pre_cor_log.txt", sep = "\t", header = T, check.names = F, row.names = 1)
rt <- t(rt)
```

```
rt <- as.data.frame(rt)
class(rt)
str(rt)
gene1name <- colnames(rt)[1]
gene2name <- colnames(rt)[2]
pdf(file="spearman_cor.pdf",
        width = 4,
        height = 4,

)
ggscatter(rt,
                x = gene1name,
                y = gene2name,
                add = "reg.line",
                conf.int = TRUE,
                cor.coef = TRUE,
                cor.method = "spearman",
                xlab = gene1name,
                ylab = gene2name,color = "blue")
dev.off()

#The logistic regression and nomogram
rt=read.table("input.txt",header=T, sep="\t",check.names=F, row.names=1)
mymodel <- glm(status~FN1 + POSTN,family=binomial(link = "logit"),data = rt)
ddist <- datadist(rt)
options(datadist="ddist")
mymodelSummary = summary(mymodel)
outTab=data.frame()
outTab=rbind(outTab,
        cbind(coef=coefficients(mymodel),
                OR=matrix(exp(coefficients(mymodel)))[,1],
                OR.95L=exp(confint(mymodel))[,"2.5 %"],
                OR.95H=exp(confint(mymodel))[,"97.5 %"],
                pvalue=mymodelSummary$coefficients[,"Pr(>|z|)"])
)
outTab=cbind(id=row.names(outTab), outTab)
write.table(outTab, file="multilogit.xls", sep="\t",row.names=F, quote=F)
rt<-read.table("input.txt",header=T,sep="\t",row.names=1)
ddist <- datadist(rt)
options(datadist="ddist")
mymodel<-lrm(status~., data=rt, x=T, y=T)
mynom<-  nomogram(mymodel,  fun=plogis,fun.at=c(0.3,0.5,0.7,0.9,0.99,0.999),lp=F,
funlabel="Risk of CRS")
pdf("Nom.pdf",8,7)
plot(mynom)
dev.off()
library(pROC)
train=read.table("pre.txt",header=T,sep="\t",check.names=F,row.names=1)
ddist <- datadist(train)
options(datadist="ddist")
test=read.table("test.txt", header=T,sep="\t",check.names=F,row.names=1)
test=test[,colnames(train)]
rt=rbind(train,test)
mymodel <- glm(status~FN1 + POSTN, family=binomial(link = "logit"), data = train)
predict <- predict.glm(mymodel,type = "response",newdata = train)
predict =ifelse(predict>0.5, 1, 0)
```

```
train$predict = predict
write.csv(train, "train_predict.csv")
true_value = train[,1]
modelroc <- roc(true_value,predict)
pdf("ROC_train_LR.pdf",6,6)
plot(modelroc, print.auc=TRUE, auc.polygon=TRUE,legacy.axes=TRUE,
     max.auc.polygon=TRUE, print.thres=TRUE,auc.polygon.col="#4169E1")
dev.off()

#Classification tree
library(rpart)
library(partykit)
library(caret)
rt <- read.table("input.txt",header=T,sep="\t",check.names = F,row.names = 1)
rt$status <-as.factor(rt$status)
tree.biop <- rpart(status ~ ., data = rt)
tree.biop$cptable
cp <- min(tree.biop$cptable[3])
prune.tree.biop = prune(tree.biop, cp <- cp)
plot(as.party(prune.tree.biop))
rparty.test <- predict(prune.tree.biop, newdata = rt,
                       type = "class")
table(rparty.test, rt$status)

#Random forest
library(ROCit)
library(randomForest)
library(rms)
library(pROC)
train_data  =  read.table("train.sva.txt",header=T,sep="\t",check.names  =  F,row.names = 1)
train_data$Group = as.factor(train_data$Group)
train_data$Group = as.factor(train_data$Group)
train_randomforest <- randomForest(Group ~.,
                                   data = train_data,
                                   ntree =500,
                                   mtry=3,
                                   importance=TRUE ,
                                   proximity=TRUE)
train_randomforest$importance
library(pROC)
pre_ran <- predict(train_randomforest,newdata=train_data)
train_data$pre_ran <- pre_ran
train_data$pre_ran <- as.numeric(train_data$pre_ran)
obs_p_ran = data.frame(prob=pre_ran,obs=train_data$Group)
table(train_data$Group,pre_ran,dnn=c("True value","Predicted value"))
```
