# Supplementary material for: Identification and external validation of the hub genes associated with cardiorenal syndrome through time-series and network analyses
Source: Aging (Albany NY). 2022 Feb 8;14(3):1351–73. doi: 10.18632/aging.203878 (PMC8876909; doi:10.18632/aging.203878)
Supplement: Supplementary Figure 1 [file aging-14-203878-s002.pdf]

SUPPLEMENTARY FIGURE

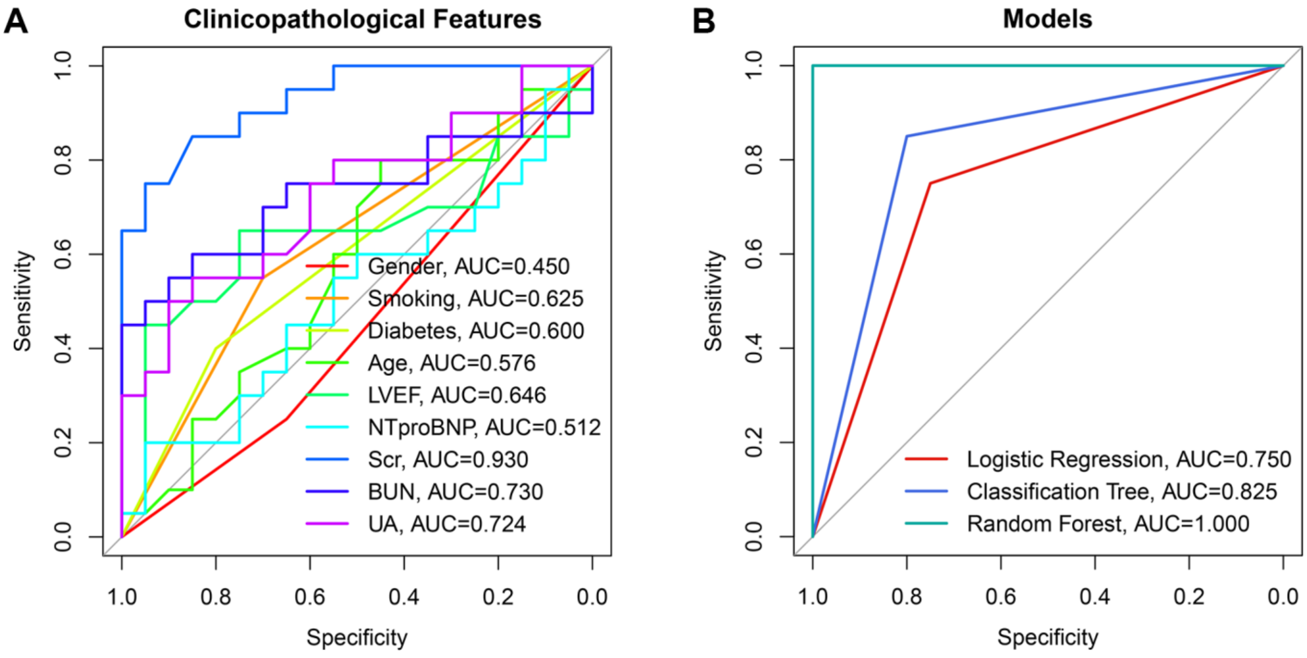

Supplementary Figure 1. The diagnosis ability of the clinicopathological parameters (**A**) and the established models (**B**).
