# Supplementary material for: Identification and external validation of the hub genes associated with cardiorenal syndrome through time-series and network analyses
Source: Aging (Albany NY). 2022 Feb 8;14(3):1351–73. doi: 10.18632/aging.203878 (PMC8876909; doi:10.18632/aging.203878)
Supplement: Supplementary Tables [file aging-14-203878-s003.pdf]

SUPPLEMENTARY TABLES

Supplementary Table 1. The PPI network analysis via CytoHubba.

Node_name	MCC	DMNC	MNC	Degree	EPC	Bottle Neck	EcCentricity	Closeness	Radiality	Betweenness	Stress	Clustering Coefficient
Kif22	6	0.46346	3	3	2.349	1	0.125	3.5	0.625	0	0	1
Pi16	1	0	1	1	2.559	1	0.25	6	1.28571	0	0	0
FN1	314	0.47886	10	10	8.878	7	0.25	11.83333	2.19643	15.56667	42	0.53333
POSTN	318	0.49882	10	10	8.793	3	0.375	12	2.25	18.23333	54	0.55556
Plod2	6	0.46346	3	3	5.435	1	0.375	8.5	1.875	0	0	1
Timp1	288	0.58315	8	8	8.423	1	0.25	10.83333	2.08929	3.9	16	0.71429
Loxl1	168	0.5854	7	7	8.365	1	0.25	10.33333	2.03571	2.66667	10	0.7619
Tnfrsf11b	2	0.30779	2	2	4.927	1	0.25	7.83333	1.76786	0	0	1
Vcan	120	0.64826	5	5	7.536	1	0.25	9.33333	1.92857	0	0	1
Thy1	24	0.56839	4	4	6.887	1	0.25	8.83333	1.875	0	0	1
Col8a1	13	0.47366	4	5	6.563	2	0.375	9.5	1.98214	26.66667	54	0.5
Mmp14	24	0.56839	4	4	6.898	1	0.25	8.83333	1.875	0	0	1
Ltbp2	24	0.56839	4	4	6.919	1	0.25	8.83333	1.875	0	0	1
Col3a1	300	0.47886	10	10	8.711	3	0.375	12	2.25	19.73333	54	0.53333
Fstl1	24	0.56839	4	4	6.665	1	0.25	8.83333	1.875	0	0	1
Col1a1	350	0.39597	13	13	9.283	2	0.375	13.5	2.41071	51.23333	112	0.39744
Prc1	12	0.47366	4	4	2.521	1	0.25	4	0.6875	0.66667	2	0.83333
Top2a	12	0.47366	4	4	2.523	1	0.25	4	0.6875	0.66667	2	0.83333
Racgap1	12	0.47366	4	4	2.59	1	0.25	4	0.6875	0.66667	2	0.83333
Cdkn3	6	0.46346	3	3	2.321	1	0.125	3.5	0.625	0	0	1

Supplementary Table 2. The raw data of the RF-qPCR experiments.

ID	Group	CT(FN1)	CT(POSTN)	CT(GAPDH)
2021016	CRS	22.714	21.62269	17.47264
2021035	CRS	21.62767	23.80968	17.20525
2021044	CRS	21.07704	22.63808	17.18994
2021045	CRS	21.36649	23.64455	16.87597
2021061	CRS	21.78654	22.12539	17.33394
2021072	CRS	19.53786	23.48876	16.93788
2021075	CRS	21.59798	22.45887	17.65224
2021079	CRS	20.83566	23.42787	16.70008
2021084	CRS	20.00147	23.39505	18.47383
2021087	CRS	21.25389	22.97611	18.36617
2021088	CRS	21.53118	23.40699	16.77462
2021096	CRS	18.06519	24.10553	16.60071
2021097	CRS	22.15883	23.26074	17.02379
2021112	CRS	21.6212	24.72875	18.31842
2021119	CRS	21.28442	21.61209	16.47575
2021131	CRS	20.43167	23.94418	18.85962

2021145	CRS	21.78432	23.92037	17.80561
2021157	CRS	20.9538	22.39475	18.02395
2021159	CRS	21.75587	22.55037	17.49426
2021165	CRS	21.72398	22.82194	17.40736
2021003	Control	22.04717	24.19026	13.92721
2021008	Control	22.29514	23.86475	16.58527
2021013	Control	22.21988	22.81359	15.47096
2021015	Control	22.8975	23.48162	16.10068
2021017	Control	22.17563	23.79683	15.873
2021021	Control	22.32354	23.76648	17.50554
2021022	Control	21.82626	23.32713	16.35305
2021026	Control	22.69801	22.79801	17.94128
2021029	Control	21.21703	23.83898	17.70134
2021031	Control	22.15845	24.01224	16.21806
2021032	Control	20.26998	21.77754	16.41206
2021034	Control	22.57254	23.01443	15.82979
2021036	Control	22.10823	25.4372	18.03494
2021038	Control	19.83442	24.09309	16.93175
2021039	Control	22.8405	23.61109	17.03301
2021040	Control	20.72439	24.23108	16.52985
2021041	Control	22.49104	22.74978	16.83147
2021042	Control	22.12857	23.5926	16.46831
2021046	Control	23.01731	22.66537	17.41212
2021047	Control	22.55935	22.80843	18.6565
